# Heuristic Search for Planning with Different Forced Goal-Ordering Constraints

**DOI:** 10.1155/2013/963874

**Published:** 2013-07-08

**Authors:** Jiangfeng Luo, Weiming Zhang, Jing Cui, Cheng Zhu, Jincai Huang, Zhong Liu

**Affiliations:** Science and Technology on Information Systems Engineering Laboratory, National University of Defense Technology, Changsha 410073, China

## Abstract

Planning with forced goal-ordering (FGO) constraints has been proposed many times over the years, but there are still major difficulties in realizing these FGOs in plan generation. In certain planning domains, all the FGOs exist in the initial state. No matter which approach is adopted to achieve a subgoal, all the subgoals should be achieved in a given sequence from the initial state. Otherwise, the planning may arrive at a deadlock. For some other planning domains, there is no FGO in the initial state. However, FGO may occur during the planning process if certain subgoal is achieved by an inappropriate approach. This paper contributes to illustrate that it is the excludable constraints among the goal achievement operations (GAO) of different subgoals that introduce the FGOs into the planning problem, and planning with FGO is still a challenge for the heuristic search based planners. Then, a novel multistep forward search algorithm is proposed which can solve the planning problem with different FGOs efficiently.

## 1. Introduction

A large majority of real-world problems have interfering subgoals. How to effectively plan for the interfering subgoals, especially when there are forced goal-ordering (FGO) constraints, has been a long term focus. As the Goal Agenda Manager (GAM) [1] used in the FF planner [2] and the ordered landmarks [3, 4] introduced in the LAMA planner [5], quite a number of approaches have been proposed but the performance results have scarcely improved. This is because, if any of the FGO constraints is violated, forward search may arrive at a deadlock, from which there is no way to reach the goal state. However, the proposed approaches such as GAM and landmark cannot detect all the deadlocks exactly and the undiscovered deadlocks make a planning difficult. In this case, this paper proposes an approach that can automatically put right the planning process when it leads the search to a deadlock and significantly improve the planning efficiency.

Many real-world problems as in military, industrial, aviation, and space domains involve FGO constraints. An example is a naval platform which has to counter many incoming missiles with different weapons [6]. Firing weapons at one missile may interfere with the interception of others. Thus it can cause the naval platform to suffer from high probability of leaking if the missiles are countered in an incorrect order. Another example is a scenario of a robot rescue [7]. Each robot has a special ability, such as survivor search/transport, cleaning barriers, or medical distribution. The rescue tasks should be finished coordinately in constrained orderings with respect to a given environment. Certain robots only care about their own subgoals and achieving them too early may result in the failure of an entire military operation. Additionally, FGO constraint can be observed in a NASA scenario as a digger robot is allowed to dig the ground on mars only after a photograph robot has taken a picture of the site [8]. Along with the complex domain dependent constraints, the FGO constraint is one of the main challenges that a planner needs to overcome for the above problems.

Next, we first give the problem statement. Some definitions and properties are proposed to explain why FGOs occur for a planning problem. Then, a novel forward search algorithm is proposed to solve the planning with FGOs. Based on the evaluation, it can be concluded that planning with FGOs is still a challenge for current automatical planners but our method can solve it efficiency.

## 2. Problem Statement

Before introducing forced goal ordering (FGO), we first give the description of a planning problem (*O*, *I*, *G*) as given in [1].

In a planning problem definition, *O* is a finite set of ground actions with the STRIPS style (in this paper). For any *o* ∈ *O*, there is *o* = (*pre*, *add*, *del*), where *pre*, *add*, and *del* are finite sets of ground atoms. *pre* is the precondition set under which the action is applicable. *add* and *del* are the atoms added or deleted after the execution of the action. *I* and *G* are the finite sets of ground atoms and represent the initial and goal state of the problem. For any given state *s* and action *o* ∈ *O*, the result of applying *o* to *s* is

(1) $$\begin{array}{c} result\left(s,o\right)=\{\begin{array}{cc} s\cup add\left(o\right)\setminus del\left(o\right), & \text{if}\;\;pre\left(o\right)\subseteq s,\\ s, & \text{otherwise}. \end{array} \end{array}$$

For the action sequence {*o*_1_, *o*_2_,…, *o*_*n*_} (*o*_*i*_ ∈ *O*, 1 ⩽ *i* ⩽ *n*), there is

(2) result(s,{o1,o2,…,on})  =result(result(s,{o1,o2,…,on−1}),on).

The planning problem is finding a sequence of actions *π*, such that *G*⊆*result*(*I*, *π*). The set of action sequence is defined as Π^*O*^. A state *s* is *reachable* from the initial state if and only if ∃*π* ∈ Π^*O*^, s.t., *s*⊆*result*(*I*, *π*). Similarly, an atom *f* is *achievable* from a certain state *s* if and only if *f* ∈ *s* or ∃*π* ∈ Π^*O*^, s.t., *f* ∈ *result*(*s*, *π*). An action *o* is *applicable* in a reachable state *s* if and only if *pre*(*o*)⊆*s*.

**Definition 1 (forced goal ordering (FGO) [1])**

For the planning problem (*O*, *I*, *G*), let *g*, *g*′ ∈ *G* be the atomic goals. We say that there is a forced ordering between *g* and *g*′, written as *g*′≺*g*, if and only if, for any state *s*(*g*, ¬*g*′), there is no plan *π* satisfying *g*′ ∈ *result*(*s*(*g*, ¬*g*′), *π*). *s*(*g*, ¬*g*′) represents a reachable state, in which *g* has just been achieved, but *g*′ is false.

In any given state, an atomic goal remaining false means that the goal is not achieved.  Definition 1 illustrates that forward search arrives at deadlock *s*(*g*, ¬*g*′) when there is *g*′≺*g* and the atomic goal *g* is achieved before *g*′. In some of the literatures [2, 9], *s*(*g*, ¬*g*′) is called a dead-end state too. During the planning process, forward search which violates any of the FGOs may lead the planning to a deadlock, from which there is no way to the goal state.

With respecting to the FGO, there is goal ordering defined as reasonable goal ordering (RGO) written as *g*′≺_*r*_ *g*. There is a reasonable ordering between *g* and *g*′, if and only if, for any reachable state *s*(*g*, ¬*g*′), there is no longer a plan that can achieve *g*′ from *s*(*g*, ¬*g*′) without deleting *g*, at least temporarily [1]. So, if there is *g*′≺_*r*_ *g* and *g* is achieved before *g*′, in order to get a plan solution, the planning must first delete the achieved goal *g*, then to achieve *g*′, and last to achieve *g* again. In this paper, as to focus the problem on FGO, it is supposed that there is no goal deletion during the planning process. Each goal cannot be deleted once it has been added.

**Figure 1:**
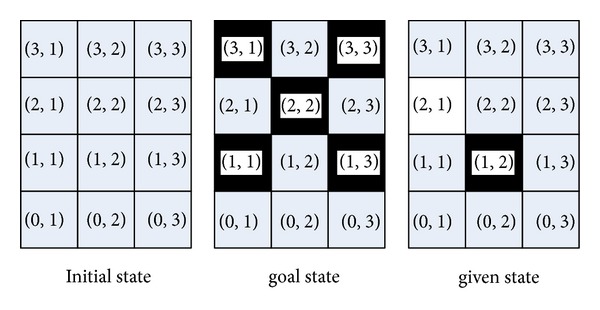
Floortile domain in IPC 2011.

The first planning domain for planning competition with FGOs is the Floortile proposed in the International Planning Competition (IPC) 2011 [10]. During the competition, no participating planner in the sequential satisficing track can solve it well.  Figure 1 shows an example of the Floortile domain in the IPC 2011. In the initial state, the status of all floor tiles is *clear*. Floor tiles need to be painted black and white, while adjacent tiles should have different colors. Robots can only paint tiles that are in front (up) or behind (down). Moreover, once a tile is painted, a robot cannot stand on it. This particular configuration makes the domain very hard to solve because of the existence of FGOs. For example, suppose a robot first selects the tile(2,1) to paint in white. In further planning steps, the robot can only stand on a tile(2,1) to paint the front tile(3,1) in black. This process can be achieved if and only if the atom (robot-at tile(2,1)) is true. However, this atom is not true and cannot be added once the tile(2,1) has been painted. The reason is that the atom (robot-at tile(2,1)) is mutually exclusive with the atomic goal (painted tile(2,1) white), and (painted tile(2,1) white) cannot be deleted once it has been added. Therefore, painting tile (2,1) before (3,1) violates the FGO constraint, in consequence, causing the search to arrive at a deadlock.

In this example, there are many FGOs in the Floortile problem, and the robots should paint tiles obeying a correct sequence. In the case of Figure 1, the FGOs are

 (*painted tile(3,1) black*) ≺ (*painted tile*(*2,1*) *white*) ≺ (*painted tile*(*1,1*) *black*);
 (*painted tile*(*3,2*) *white*) ≺ (*painted tile*(*2,2*) *black*) ≺ (*painted tile*(*1,2*) *white*);
 (*painted tile*(*3,3*) *black*) ≺ (*painted tile*(*2,3*) *white*) ≺ (*painted tile*(*1,3*) *black*).

In the above domain, all the FGOs exist in the initial state. No matter which approach is adopted to achieve an atomic goal, all the atomic goals should be achieved in a given sequence starting from the initial state. Otherwise, the planning may arrive at a deadlock. However, in some real-world planning problem, there is no FGO in the initial state. For any *g*, *g*′ ∈ *G*, planning starting from the initial state to firstly achieve *g* or *g*′ would not lead to a deadlock. However, the planning may arrive at a given state *s*(¬*g*_1_, ¬*g*_2_, ¬*g*_3_) (*g*_1_, *g*_2_, *g*_3_ ∈ *G*) without FGO among *g*_1_, *g*_2_, and *g*_3_. Now, if certain plan as *π*_1_ is selected to achieve *g*_1_ while translating the search to state *s*(*g*_1_, ¬*g*_2_, ¬*g*_3_), then there is *g*_2_≺*g*_3_ or *g*_3_≺*g*_2_.

**Figure 2:**
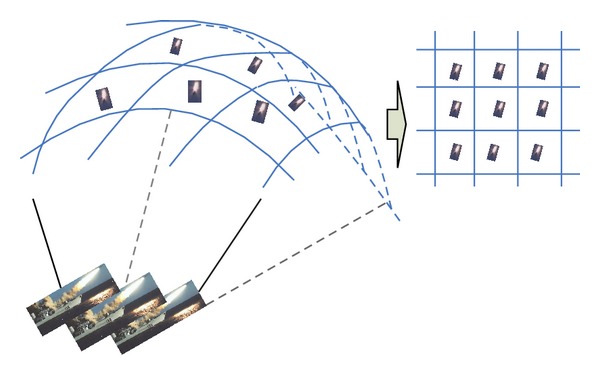
Air defense of a naval group.

Figure 2 shows an example for the air defense of a naval group (ADoNG). A naval group has some Surface to Air Missile (SAMs) and chaffs to intercept the incoming antiship missiles. The arrived antiship missiles at the same time is supposed to locate at a spherical surface above the naval group, which can be transferred into a *m* × *n* rectangular plane as shown in Figure 2. In each rectangle, there is an antiship missile, while one ship of the naval group can fire a SAM or chaff to intercept it. However, once a chaff is employed to intercept an antiship missile in a given rectangle, the rectangles in the up, down, left, and right of the given rectangle should be interfered by the chaff cloud. If certain rectangle is interfered by the chaff cloud from one direction, the antiship missile in this interfered rectangle can only be intercepted by chaff, because chaff cloud can prevent the radar from guiding the SAM interception. Moreover, if certain rectangle is interfered by the chaff cloud from more than one direction, the antiship missile in this rectangle cannot be intercepted, because the radar of the naval group may lose the accurate position of the antiship missile. The goal state is to intercept all the incoming missiles by the given SAMs and chaffs.

**Figure 3:**
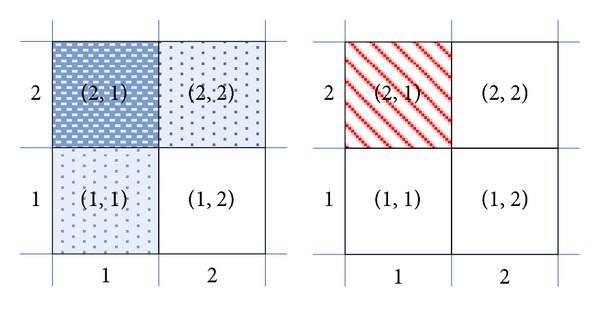
Air defense of naval group.

For the planning of the air defense of a naval group, there is no FGO in the initial state. Taking the problem shown in Figure 3 as an example, there are 4 antiship missiles in a 2 × 2 rectangular plane with 3 chaffs and 1 SAM. Obviously, in the initial state, every antiship missile can be intercepted with the highest priority. However, if the antiship missile in rectangle (2, 1) is firstly intercepted by a chaff, there are FGOs (1,2)≺(1,1) and (1,2)≺(2,2) in the successor state, and the antiship missile in (1, 2) must be intercepted by a SAM. However, if the antiship missile in rectangle (2, 1) is firstly intercepted by a SAM, there is no FGO for the remaining antiship missiles in (1,1), (1,2), and (2,2).

## 3. Related Works

Heuristic search planning (HSP) has become a dominant domain independent paradigm over the last decade [5]. The HSP method, first proposed by Bonet and Geffner [11], performs a forward search from an initial state to a goal state in a search graph. This method employs some powerful heuristic estimators to guide the search in a fast forward manner towards the goal state, with the help of heuristics for choosing helpful actions to extend the search closer to the goal state. In the past, great success has been achieved in heuristic search planning systems, such as FF [2], FD [12], SGPlan [13], and LAMA [5]. Researchers have also considered various goal interactions in multiple-goal achievement and detection [1, 14]. All of the above planners have their own approaches to deal with the goal orderings.

Among the previous works, the most relevant methods to our approach are Goal Agenda Manager (GAM) and ordering landmarks. The concept of a Goal Agenda Manager (GAM) was proposed in [1] to detect the reasonable goal orderings. The GAM is widely used in many planning systems such as IPP and FF, which can improve the performances of IPP and FF dramatically. A GAM defines the order in which the subgoals are achieved. In the beginning of a search process, a GAM is employed to check all the ordering relationships of each atomic goal pair. Then, the search divides the goal set into many subsets so that the planner can achieve each of them in sequence.

For each atomic goal pair as *g*, *g*′ ∈ *G*, GAM uses *F*_*Dg*_^*g*^ = ⋂_*o*∈*O*, *g*∈*add*(*o*)_ *del*(*o*) and O* = *O*_*g*_∖{*o* ∈ *O* | *pre*(*o*)∩*F*_*Dg*_^*g*^ = *∅*}, where *O*_*g*_ = {*o* ∈ *O* | *g* ∉ *del*(*o*)}, to calculate whether there is an action sequence *p* ∈ *P* ^O*^ satisfying *g*′ ∈ *result*(*s*(*g*, ¬*g*′), *p*). If there is not, there exists an ordering defined as *g*′ ≺_*r*_ *g*. Otherwise, the GAM checks whether *g*≺_*r*_ *g*′ exists. Therefore, there are *P*_|*G*|_^2^ atomic goal pairs that need to be checked.

A concept known as “landmark” is defined to extend the GAM on goal ordering at top-level atomic goals as well as certain states known as landmarks during a planning process [3, 4]. For a planning problem (*O*, *I*, *G*), an atom *l* is called a landmark if, for any *p* = {*o*_1_, *o*_2_,…, *o*_*n*_} ∈ *P* ^*O*^ and *G*⊆*result*(*I*, *p*), there is *l* ∈ *result*(*I*, {*o*_1_,…, *o*_*i*_}) (1 ⩽ *i* ⩽ *n*). A planning system that uses landmarks obtains all the landmarks of the problem at the beginning of the planning process. The planner then orders them heuristically. It uses a backtracking method via a relaxed plan graph (RPG) [2] to find the candidate landmarks and their orders. For example, all the atomic goals are landmarks. For each atomic goal *g*, the atoms in ⋂_∀*o*∈*O*, *g*∈*add*(*o*)_ *pre*(*o*) are treated as new landmarks, where there is relationship *f*≺_*r*_ *g* for each *f* ∈ ⋂_∀*o*∈*O*, *g*∈*add*(*o*)_ *pre*(*o*). Then, *f* is treated as a new atomic goal. The landmark-generation algorithm repeats the above process from the top level of the RPG to the lowest level.

**Figure 4:**
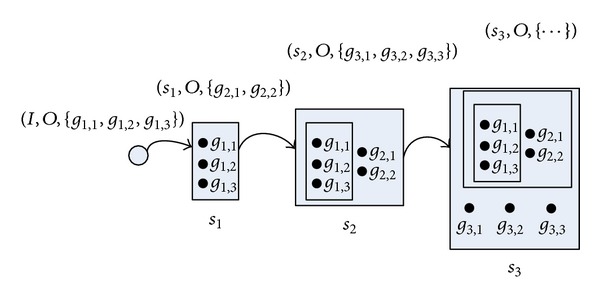
The incremental planning process.

The third method to handle the goal ordering is the incremental planning process adopted by the planner SGPlan6 [13, 15]. As shown in Figure 4, the incremental planning process try to check part of the goal orderings in the initial state. Some of the checked atomic goals which can be achieved with high priority are firstly handled. Then, the incremental planning process try to find out more other goal orderings in current state and achieve part of atomic goals with high priority. The planning continues the above process until all the atomic goals are achieved.

## 4. Why FGOs Occur?

**Definition 2 (goal achievement operation (GAO))**

For planning problem (*O*, *I*, *G*), *o* ∈ *O* is a GAO, if ∃*g* ∈ *G*, satisfying *g* ∈ *add*(*o*). *o* is written as *o*_*g*_.

**Definition 3 (available GAO)**

*o*_*g*_ is available in a given state *s*, if and only if *pre*(*o*_*g*_)⊆*s*, or ∃*π* ∈ Π^*O*^, s.t.   *pre*(*o*_*g*_)⊆*result*(*s*, *π*).

For an unachieved atomic goal *g* in *s*, an available GAO as *o*_*g*_ represents a plan, written as *π*_*o*_*g*__, while *g* ∈ *result*(*s*, *π*_*o*_*g*__). So, if *pre*(*o*_*g*_)⊆*s*, there is *π*_*o*_*g*__ = {*o*_*g*_}. Otherwise, if *pre*(*o*_*g*_)⊆*result*(*s*, *π*), there is *π*_*o*_*g*__ = {*π*, *o*_*g*_}.

**Definition 4 (available GAO sequence)**

Suppose that *G*′⊆*G* while *s*(¬*G*′) is reachable, and there is at least one available GAO as *o*_*i*_ ∈ *O* (1 ⩽ *i* ⩽ |*G*′|) for each *g*_*i*_ ∈ *G*′ in *s*(¬*G*′) (|*G*′| is the number of atomic goals contained in *G*′). These GAOs can be ranked as an available GAO sequence if and only if

(1) these GAOs are ranked in a correct sequence {*o*_*j*_1__, *o*_*j*_2__,…, *o*_*j*_|*G*′|__}, (*j*_*i*_ = 1,2,…, |*G*′|, *j*_*k*_ ≠ *j*_*l*_ if *k* ≠ *l*, 1 ⩽ *i*, *k*, *l* ⩽ |*G*′|);
(2) there are number of |*G*′| corresponding plans {*π*_*j*_1__, *π*_*j*_2__,…, *π*_*j*_|*G*′|__} such that
  (3) $$\begin{array}{c} pre\left(o_{j_{1}}\right)\subseteq result\left(s\left(\neg G^{\prime }\right),\pi_{j_{1}}\right),\\ g_{j_{1}}\in result\left(s\left(\neg G^{\prime }\right),\left\{\pi_{j_{1}},o_{j_{1}}\right\}\right)=s_{1},\\ pre\left(o_{j_{2}}\right)\subseteq result\left(s_{1},\pi_{j_{2}}\right),\\ \left\{g_{j_{2}},g_{j_{1}}\right\}\subseteq result\left(s_{1},\left\{\pi_{j_{2}},o_{j_{2}}\right\}\right)=s_{2},\\ \vdots \\ pre\left(o_{j_{\left|G^{\prime }\right|}}\right)\subseteq result\left(s_{|G^{\prime }|-1},\pi_{j_{\left|G^{\prime }\right|}}\right),\\ G^{\prime }\subseteq result\left(s_{|G^{\prime }|-1},\left\{p_{j_{\left|G^{\prime }\right|}},o_{j_{\left|G^{\prime }\right|}}\right\}\right). \end{array}$$

**Property 1**

For the planning problem (*O*, *I*, *G*) with FGO constraints, the reachable state *s* is not a deadlock if and only if there is at least an available GAO sequence for the maximum unachieved atomic goal set in *s*.

**Proof**

Based on Definition 4, suppose that the maximum unachieved atomic goal set in *s* is *G*′. Since goal deletion is not considered in this paper, there is a plan

(4) $$\begin{array}{c} \pi =\left\{\pi_{j_{1}},o_{j_{1}},\pi_{j_{2}},o_{j_{2}},\ldots ,\pi_{j_{\left|G^{\prime }\right|}},o_{j_{\left|G^{\prime }\right|}}\right\}, \end{array}$$

s.t., *G*⊆*result*(*s*(¬*G*′), *π*). Therefore, *s*(¬*G*′) is not a deadlock. Additionally, if *s* is not a deadlock, there must be at least one available GAO sequence in *s*.

**Definition 5 (excludable constraint of GAO)**

For a reachable state *s*(¬*g*_1_, ¬*g*_2_), there are available GAO *o*_*g*_1__ for *g*_1_ and *o*_*g*_2__ for *g*_2_. There is an excludable constraint in *s*(¬*g*_1_, ¬*g*_2_), written as *o*_*g*_1__↛*o*_*g*_2__, if and only if *o*_*g*_2__ is unavailable in state *result*(*s*(¬*g*_1_, ¬*g*_2_), *π*_*o*_*g*_1___).

**Definition 6 (excludable GAO set)**

In a reachable state *s*(¬*G*′)  (*G*′⊆*G*), the excludable GAO set for the available GAO *o*_*g*_ (*g* ∈ *G*′) is written as ${\hat{O}}_{o_{g}}(s(\neg G^{\prime }))$, if and only if, (1) for any *o*_*g*_↛*o*_*g*′_  (*g*′ ∈ *G*′,  *g*′ ≠ *g*), there is $o_{g^{\prime }}\in {\hat{O}}_{o_{g}}(s(\neg G^{\prime }))$; (2) for any $o_{g^{\prime }}\in {\hat{O}}_{o_{g}}(s(\neg G^{\prime }))$, there is *o*_*g*_↛*o*_*g*′_; (3) for any $o_{g^{\prime }}\notin {\hat{O}}_{o_{g}}(s(\neg G^{\prime }))$, there is not *o*_*g*_↛*o*_*g*′_.

**Definition 7 (equivalent GAO)**

In a reachable state *s*(¬*g*)  (*g* ∈ *G*), *o*_*g*_^1^ and *o*_*g*_^2^ are two different available GAOs for *g*. *o*_*g*_^1^ and *o*_*g*_^2^ are equivalent in *s*(¬*g*), written as *o*_*g*_^1^≅*o*_*g*_^2^, if and only if ${\hat{O}}_{o_{g}^{1}}(s(\neg g))={\hat{O}}_{o_{g}^{2}}(s(\neg g))$.

**Definition 8 (equivalent State)**

Reachable states *s*_1_ and *s*_2_ are equivalent, written as *s*_1_≅*s*_2_, if and only if, for any *π* ∈ Π^*o*^, there is *G*⊆*result*(*s*_1_, *π*), there must be *G*⊆*result*(*s*_2_, *π*) and vice versa.

In a reachable state *s*(¬*g*), for an available GAO *o*_*g*_ with pre(og) ⊆ s(¬g), there may be two different action sequences *π*_1_ and *π*_2_ satisfying *pre*(*o*_*g*_)⊆*result*(*s*(¬*g*), *π*_1_) and *pre*(*o*_*g*_)⊆*result*(*s*(¬*g*), *π*_2_). However, *result*(*s*(¬*g*), *π*_1_) might not be equivalent with *result*(*s*(¬*g*), *π*_2_). In this case, the atomic goal *g* achieved by the same GAO *o*_*g*_ may lead the search to two different nonequivalent states. Planning with this feature may increase the search space dramatically when handling the planning with FGOs. Therefore, with respect to the above case, we can define two different GAOs as *o*_*g*_^*π*_1_^ and *o*_*g*_^*π*_2_^ to replace *o*_*g*_ to ensure that, in a given state, achieving an atomic goal by the same GAO should lead the search to the equivalent state. Namely, for a given reachable state *s*(¬*g*) and an available GAO *o*_*g*_, for any *π*_1_, *π*_2_ ∈ Π^*O*^, if there are *pre*(*o*_*g*_)⊆*result*(*s*(¬*g*), *π*_1_) and *pre*(*o*_*g*_)⊆*result*(*s*(¬*g*), *π*_2_), there must be *result*(*s*(¬*g*), {*π*_1_, *o*_*g*_})≅*result*(*s*(¬*g*), {*π*_2_, *o*_*g*_}).

**Property 2**

For a reachable sate *s*(¬*G*′) (*G*′⊆*G*, *g* ∈ *G*′) and two available GAOs *o*_*g*_^1^ and *o*_*g*_^2^, there are *s*_1_ = *result*(*s*(¬*G*′), *π*_*o*_*g*_^1^_) and *s*_2_ = *result*(*s*(¬*G*′), *π*_*o*_*g*_^2^_). If both *s*_1_ and *s*_2_ are not deadlock and there is *o*_*g*_^1^≅*o*_*g*_^2^, there must be *s*_1_≅*s*_2_.

**Proof**

Suppose that *O*_*G*′_ is the set of all possible available GAO sequences contained in *s*(¬*G*′), *O*_*G*′∖*g*_^1^ is the set of all possible available GAO sequences contained in *s*_1_, and *O*_*G*′∖*g*_^2^ is the set of all possible available GAO sequences contained in *s*_2_. As *o*_*g*_^1^ and *o*_*g*_^2^ have the same excludable GAO set, there must be *O*_*G*′_∖*O*_*G*′∖*g*_^1^ = *O*_*G*′_∖*O*_*G*′∖*g*_^2^. It can be inferred that *O*_*G*′∖*g*_^1^ = *O*_*G*′∖*g*_^2^. As *s*_1_ and *s*_2_ have the same set of possible available GAO sequences, it can be declared that, for any available GAO sequence contained in *s*_1_(*s*_2_), this available GAO sequence must be available in *s*_2_(*s*_1_). So there is *s*_1_≅*s*_2_.

**Property 3**

For the planning problem (*O*, *I*, *G*), *s*(¬*g*) is a reachable state. *o*_*g*_^1^ and *o*_*g*_^2^ are two different available GAOs of *g* in *s*(¬*g*) while *o*_*g*_^1^≅*o*_*g*_^2^. Starting from *s*(¬*g*), if selecting *o*_*g*_^1^ to achieve *g* leads the planning to state *s*_1_ and selecting *o*_*g*_^2^ leads the planning to state *s*_2_, then, for any ¬*g*_1_, ¬*g*_2_ ∈ *s*_*i*_ (*i* = 1,2), if there is *g*_1_≺*g*_2_ in *s*_1_(*s*_2_), there must be *g*_1_≺*g*_2_ in *s*_2_(*s*_1_).

**Proof**

Based on the Property 2, it can be inferred that *s*_1_ and *s*_2_ have the same set of possible available GAO sequences. So if there is *g*_1_≺*g*_2_ in *s*_1_, there is no available GAO sequence contained in *s*_1_ which can achieve *g*_2_ before *g*_1_. As *s*_1_ and *s*_2_ have the same set of possible available GAO sequences, so starting from *s*_2_, *g*_2_ cannot be achieved before *g*_1_ too. So there is *g*_1_≺*g*_2_ in *s*_2_. Obviously, by the same way, it can be inferred that, if there is *g*_1_≺*g*_2_ in *s*_2_, there must be *g*_1_≺*g*_2_ in *s*_1_.

Property 3 illustrates that, during the planning process, selecting the equivalent GAO to achieve an atomic goal introduces the same possible FGOs into the planning.

**Definition 9 (independent goal set)**

For the planning problem (*O*, *I*, *G*), the goal set *G* can be divided into *k* independent goal sets, written as {*G*_1_, *G*_2_,…, *G*_*K*_} (1 ⩽ *k*), while *G*_1_ ∪ *G*_2_ ∪ ,…, *G*_*k*_ = *G* and *G*_*i*_∩*G*_*j*_ = *∅* (for all 1 ⩽ *i*, *j* ⩽ *k*). *G*_*i*_ and *G*_*j*_ are called independent with each other, if and only if, for any *g*_*i*_ ∈ *G*_*i*_ and *g*_*j*_ ∈ *G*_*j*_, there is no excludable constraint between the GAO of *g*_*i*_ and *g*_*j*_ in each reachable state.

Based on the above discussion, it can be concluded that FGOs occur just because there are excludable constraints among the GAOs of different atomic goals. Take the instance shown in Figure 1 as a example. In the initial state, the available GAO for atomic goal *(painted tile(3,1) black)* is *(paint-up robot1 tile(3,1) tile(2,1))*. The available GAOs for atomic goal *(painted tile(2,1) white)* are *(paint-up robot1 tile(2,1) tile(1,1))* and *(paint-down robot1 tile(2,1) tile(3,1))*, as there are

 
*(paint-up robot1 tile(2,1) tile(1,1))*  ↛  *(paint-up robot1 tile(3,1) tile(2,1))*;
 
*(paint-down robot1 tile(2,1) tile(3,1))*  ↛  *(paint-up robot1 tile(3,1) tile(2,1))*;
 So there is *(painted tile(3,1) black)*  ≺  *(painted tile(2,1) white)* in the initial state.

Furthermore, for the planning which has no FGO in the initial state, the excludable constraints among the GAOs may introduce FGOs into the planning process. As the example shown in Figure 3, in the initial state, each antiship missile can be intercepted by a SAM or chaff. However, as there are

 
*(Chaff-Intercept (2,1))*  ↛  *(SAM-Intercept (1,1)) *
 
*(Chaff-Intercept (2,1))*  ↛  *(SAM-Intercept (2,2)) *
 the FGOs *(intercept (1,2))*  ≺  *(intercept (1,1))* and *(intercept (1,2))*  ≺  *(intercept (2,2))* occur.

Obviously, for the planning problem (*O*, *I*, *G*), if for all *g*_*i*_, *g*_*j*_ ∈ *G* (*i* ≠ *j*) while *g*_*i*_ is independent with *g*_*j*_ as Definition 9 described, no FGO may occur during the planning process.

Next, a forward search algorithm is proposed based on the above discussion to solve the planning problem with different FGOs.

## 5. A Novel Forward Planning Algorithm

During the planning process, selecting a GAO with the bigger excludable GAO set to achieve an atomic goal has the higher probability to introduce FGOs into the planning. Generally, for the same atomic goal, a search algorithm prefers to select the GAO with the smaller excludable GAO set first to achieve it. However, for some planning problem, keeping to select the GAO with smaller excludable GAO set first may cause certain operation resource excessively consumed. In this case, the later planning process can only select the GAO with the bigger excludable GAO set to achieve each atomic goal. Then, planning may lead to a deadlock as the FGOs introduced by the excludable GAOs. Therefore, with respect to the planning algorithm proposed in this paper, the atomic goal as *g*_1_ with the fewest number of available GAO is firstly selected to be achieved by an available GAO with the biggest excludable GAO set. Then, calculate the number of available GAO for the remaining unachieved atomic goals. The atomic goal, whose number of available GAO is decreased after the achievement of *g*_1_, is selected to be achieved with high priority. Continuing the above process, if planning arrives at a state which contains an unachieved atomic goal without available GAO, move the achievement sequence of this atomic goal ahead and find an available GAO with the smallest excludable GAO set to achieve it, while ensuring that all the prior achieved goals can also be achieved by the prior selected GAOs (or the equivalent GAOs).

The excludable GAO set based forward search algorithm *Ex_MsFS* (multi-step forward search) for the planning with FGOs is displayed in Algorithm 1. In the initial state *s*, the planning selects an atomic goal with the fewest number of available GAO to be achieved first (step 04). With respect to the selected atomic goal as *g*, the available GAO as *o*_*g*_ with the biggest excludable GAO set is selected with the highest priority to achieve *g* (step 08). Then, in the successor state *s*′ after *g* achieved (step 09), there exist the following two cases. In case one, there is an unachieved atomic goal as *g*′, which has no available GAO in *s*′. In this case, *s*′ is a deadlock. All possible available GAOs for *g*′ in *s*′ are excluded by the GAOs which have been adopted to achieve the earlier selected atomic goals contained in *L*. So the achievement sequence of *g*′ should be moved ahead (step 12). The detailed moving algorithm is lately discussed in Algorithm 2. For case two (step 20), if all unachieved goals in *s*′ have available GAO, the atomic goals which have fewer available GAOs in *s*′ than that in *s* should be selected to be achieved with high priority starting from *s*′ by the depth-first search strategy (steps 23, 07).

The algorithm *Move*_*ahead* used in step 12 of Algorithm 1 is displayed in Algorithm 2. For the step 11 of Algorithm 1, when there is an atomic goal as *g* having no available GAO in current state, it means that certain achieved goals stored in list *L* (step 09 of Algorithm 1) should not be achieved before *g*. So the achievement sequence of *g* should be tried to move ahead. Suppose there are *num* elements contained in *L*. Each element is written as (*s*′, *o*_*g*_, *s*), which means that selecting the available GAO *o*_*g*_ in current state *s* to achieve goal *g* transfers the state to the successor *s*′. The atomic goals contained in the list *L* are achieved by the sequence from the head to the end. The algorithm tries to set *g* as the *i*th (1 ⩽ *i* < *num*) goal to be achieved (step 02). The atomic goals stored in *L* from the location index 1 to *i* − 1 are still achieved by the previously selected GAOs (steps 04–06). Then, it is the turn to select an available GAO to achieve *g*. As to ensure that all the atomic goals stored in list *L* from the location index *i* to *num* can still be achieved by its previously selected GAOs or the equivalent GAOs of the previously selected GAOs, the algorithm chooses an available GAO with the smallest excludable GAO set to achieve *g* (step 09–17). Now, the algorithm starts to check whether the atomic goals stored in list *L* from the location index *i* to *num* can still be achieved by its previously selected GAOs or whose equivalent GAOs (steps 21–35). If it is, it means that, with respect to the atomic goals in *L*, *g* can be taken as the *i*th goal to be achieved. So the algorithm returns *true* (steps 31–33). Otherwise, move *g* ahead as the (*i* − 1)th goal to be achieved. If achieving *g* before all the atomic goals in list *L* still cannot ensure that the atomic goals stored in list *L* can be achieved by their previously selected GAOs or whose equivalent GAOs, the *Move_ahead* algorithm returns *false* (step 36).

Based on Algorithms 1 and 2 and the Definition 9, it can be inferred that, for each do_while loop (steps 06–24) of the algorithm *Ex_MsFS*, the achieved goals contained in each list *L* defined in step 03 of Algorithm 1 come from the same independent goal set. During the depth-first search process, all the atomic goals which are related with each selected goal (in step 04 of Algorithm 1) are stored in each list *Q*. Obviously, the atomic goals in each list *Q* are from the same independent goal set. For the moving ahead process displayed in Algorithm 2, the requirement that all the achieved goals in each list *L* can still be achieved by their previously selected GAO or their equivalent GAO after the achievement sequence of certain atomic goal is moved ahead, is to ensure that with respect to each list *L*, the elements having contained in list *Q* would not be changed during the moving ahead process. The reason is that the equivalent GAO makes an atomic goal lose the same number of available GAOs. So the depth-first process could not be inferred by the moving ahead process.

**Figure 5:**
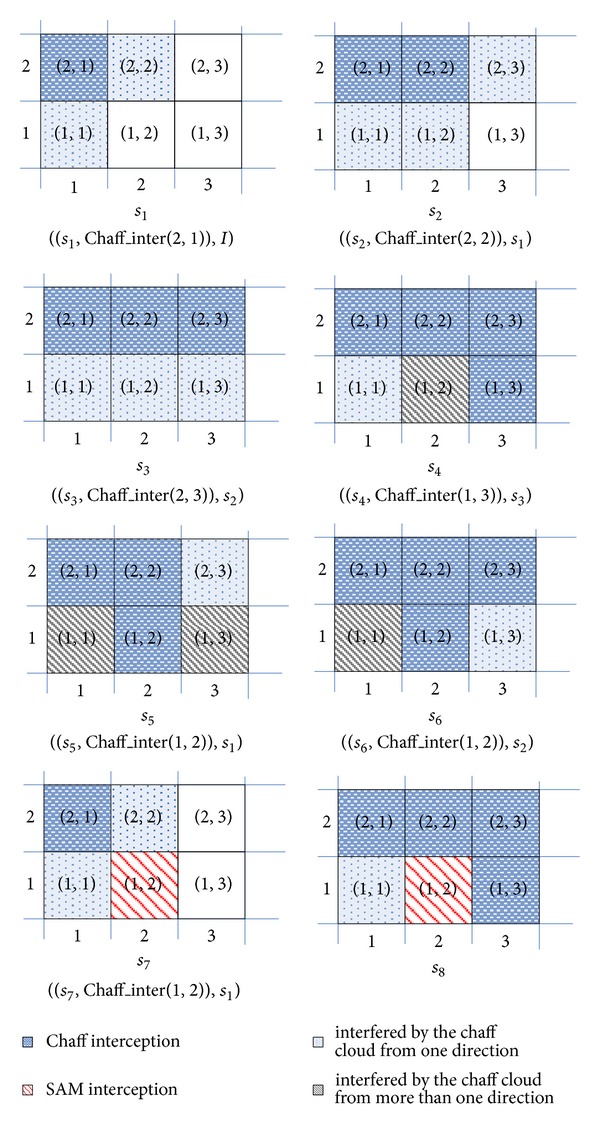
The example of air defense for a naval group.

Figure 5 gives an example of the air defense for a naval group to illustrate the search process of *Ex_MsFS*. In the initial state, there are 6 antiship missiles. There are 5 chaffs and a SAM that can be used to intercept all the antiship missiles while the antiship missile in rectangle (2,1) must be intercepted by a chaff. As there is only one available GAO (*Chaff*_*Inter*(2,1)) for the missile in (2,1), *Ex_MsFS* selects the missile in (2,1) to firstly intercept by a chaff and transfers the state to *s*_1_ (step 04 of Algorithm 1). Element (*s*_1_, *Chaff*_*Inter*(2,1), *I*) is pushed into list *L* (steps 08-09 of Algorithm 1). When the missile in (2,1) is intercepted by a chaff, the rectangles (1,1) and (2,2) are interfered by the chaff cloud coming from rectangle (2,1). So the missiles in (1,1) and (2,2) cannot be intercepted by SAM. The number of available GAOs for missiles in (1,1) and (2,2) in *s*_1_ is fewer than that of in *I*. Therefore, missiles in (1,1) and (2,2) are pushed back into list *Q* (steps 22-23 of Algorithm 1) and assigned the higher priority to be intercepted during in the depth-first process. Suppose that missile in (2,2) is firstly selected to be intercepted by a chaff in state *s*_1_ (step 07). Planning leads to *s*_2_ and element (*s*_2_, *Chaff*_*Inter*(2,2), *s*_1_) is pushed back into list *L* (step 09 of Figure 4). At the same time, missiles in (1,2) and (2,3) are pushed back into list *Q* (step 22 of Algorithm 1).

Continuing the above process, element (*s*_3_, *Chaff*_*Inter*(2,3), *s*_2_) is pushed back into list *L* and missiles in (1,3) is pushed back into list *Q*. Now, the planning arrives at state *s*_3_ and list *Q* pops back the missile in (1,3) to intercept. As rectangle (1,3) is interfered by the chaff cloud from rectangle (2,3), the missile in (3,1) can only be intercepted by a chaff. In this case, rectangle (2,3) should be interfered by the chaff cloud from rectangles (2,2) and (1,3) as the state *s*_4_ shows. So there is no available GAO for the missile in (1,3), and the interception sequence of the missile in (1,3) needs to be moved ahead (steps 11-12 of Algorithm 1).

Now, the *move_ahead* algorithm tries to intercept the missile in (1,2) before that of in (1,3). It means to intercept the missile in (1,2) using (*Chaff*_*Inter*(1,2)) starting from state *s*_3_. This selection may lead the search to state *s*_5_, in which missiles in (1,1) and (1,3) cannot be intercepted. So the intercept sequence of missile in (1,2) should be further moved ahead. The *move_ahead* algorithm tries to intercept it before the missile in (2,3), which leads the planning to state *s*_6_. Obviously, the missile in (1,1) cannot be intercepted starting from *s*_6_. So the missile in (1,2) should be intercepted before the missile in (2,2). It means intercepting the missile in (1,2) starting from *s*_1_. Now, the available GAO (*SAM*_*Inter*(1,2)) with the smaller excludable GAO set is selected. Furthermore, all the missiles in (2,2), (2,3), and (1,3) can still be intercepted by its previously selected GAOs. After the moving ahead process, planning arrives at state *s*_8_, from which the goal state can be arrived after the missile in (1,1) is intercepted by the last one chaff.

## 6. Discussion of the Problem and Algorithm

This section proposes some properties about the planning with FGOs and the search algorithm *Ex_MsFS*.

**Property 4**

The complexity for solving the planning with FGOs, all of which exist in the initial state and are irrelevant with the approach for each atomic goal to be achieved, is P_*n*_^*n*^, where *n* = |*G*|.

**Proof**

As all the FGOs exist in the initial state and are irrelevant to the approach for each atomic goal to be achieved, the search can arrive at the goal state if and only if all the atomic goals are achieved by a correct sequence. It is a complete permutation problem. So the complexity is P_*n*_^*n*^.

**Property 5**

The complexity for solving the planning problem, in which there is no FGO in the initial state but FGOs would occur during the planning process if and only if certain goal is achieved by an inappropriate approach, is up to P_*n*_^*n*^ · *K* ^*n*^, where *n* = |*G*| and *K* is the average number of approach that can be adopted to achieve each atomic goal.

**Proof**

In this case, all the atomic goals should be achieved in a correct sequence, and each atomic goal should be achieved by an appropriate approach. However, as there is no FGO in the initial state, not all the atomic goals need to take part in the complete permutation. So the complexity is up to P_*n*_^*n*^ · *K* ^*n*^.

For the Floortile problem, the solving complexity is P_*n*_^*n*^ while the air defense planning problem for a naval group is up to P_*n*_^*n*^ · *K* ^*n*^.

**Property 6**

For a solvable problem with FGOs, the *Ex_MsFS* algorithm is sufficient to returning a plan solution if the *move_ahead* algorithm returns *true* (step 12 of Algorithm 1) for each time it is called.

**Proof**

If the *move_ahead* algorithm returns *true* (step 12 of Algorithm 1) for each time that it is called, it means the search process which violates certain FGO constraints has been put right. So the search process can arrive at the goal state and return a plan solution.

For some planning problem, the search may arrive at certain state, from which no matter which, atomic goal is firstly selected to achieve, the search should arrive at a deadlock. In this case, the *move_ahead* algorithm always returns *false* and the *Ex_MsFS* algorithm cannot return the plan solution. Therefore, further works need to be done to extend the *Ex_MsFS* algorithm to a more general case.

**Property 7**

Evaluated by the Relaxed Graph [2], *Ex_MsFS* algorithm is an enforce hill-climbing algorithm, which climbs multiple steps each time.

**Proof**

Suppose *H*(*s*) is the distance, calculated by the Relaxed Plan Graph, between the reachable state *s* and the goal state *G*, where *H*(*G*) = 0. In the estimator of Relaxed Graph,

(5) $$\begin{array}{c} H\left(s\right)=h_{g_{1}}\left(s\right)+h_{g_{2}}\left(s\right)+\cdots +h_{g_{n}}\left(s\right)-\underset{O_{s}}{\sum }\left(\left|o\right|-1\right), \end{array}$$

where *g*_*i*_ ∈ *G* (1 ⩽ *i* ⩽ *n*) is the unachieved atomic goal in *s*, and *h*_*g*_*i*__(*s*) is the number of actions in the relaxed graph to achieve the atomic goal *g*_*i*_ starting from *s*. Set *O*_*s*_ contains actions shared by different atomic goals during their achievements in the relaxed graph, where |*o*| is the frequency of *o* that has been shared. For each atomic goal as *g*_*i*_ selected in step 04 of Algorithm 1, the *Ex_MsFS* algorithm selects an available GAO and generates a plan to achieve *g*_*i*_, which transfers the search from the current state *s* to the successor state *s*′. As action deletion is not considered in the relaxed graph, there are *h*_*g*_*i*__(*s*′) = 0 and

(6) $$\begin{array}{c} H\left(s\right)-H\left(s^{\prime }\right)=h_{g_{i}}\left(s\right)-\left(\underset{O_{s}}{\sum }\left(\left|o\right|-1\right)-\underset{O_{s^{\prime }}}{\sum }\left(\left|o\right|-1\right)\right). \end{array}$$

Suppose that *g*_*i*_ has *k* independent actions to other atomic goals in the Relaxed Graph starting from *s*. The value of ∑_*O*_*s*__(|*o* | −1) − ∑_*O*_*s*′__(|*o* | −1) is *h*_*g*_*i*__(*s*) − *k*. Therefore, for each selected atomic goal, there is *H*(*s*) − *H*(*s*′) = *k*. The search direction of the *Ex_MsFS* algorithm is along the direction of the enforced hill climbing by *k* steps at a time toward a goal state.

## 7. Evaluation

This paper evaluates the proposed algorithm *Ex_MsFS* by comparing it with the following planning systems:

1. FF planning system [2], in which the GAM heuristic is used for the detection of goal ordering,
2. SGPlan6 [13], which adopts the incremental planning process to handle the goal orderings,
3. LAMA 2008 planning system [5], which adopts the hill-climbing strategy where the preferred actions of each step are selected by the FF and landmark heuristics. Moreover, the orderings of (disjunction) landmarks are calculated based on the domain transition graph and the causal graph.

FF, SGPLan6, and LAMA 2008 won the 1st prize of the satisficing planning track in IPC 2001, 2006, and 2008 respectively. In addition, the LAMA 2008 planning system consists of translating, processing, and searching modules. The translating and processing modules are used to construct some structured data, based on which, searching module is employed for forward search. In this paper, in order to compare the *Ex_MsFS* with the previous approaches, we use the *Ex_MsFS* algorithm to replace the search modules of the Lama 2008 planning system.

The competition domains are the Floortile proposed in IPC 2011 and the air defense of naval group problem introduced in Section 1. For an air defense of naval group problem, there are *m* × *n* antiship missiles while |(*m* × *n*)/3| SAMs and *m* × *n* − |(*m* × *n*)/3| chaffs can be used. As this paper focuses on the efficiency when to solve a planning problem with FGOs, the quality of the planning solution is not considered. So we evaluate each planner by the scale of planning problems it can solve. The experiments are implemented in the Mac OS X with 2.4 GHz Intel Core 2 Duo and 2 GB 1067 MHz DDR3 memory. Each instance fails if the running time is more than 1,200 seconds.

**Figure 6:**
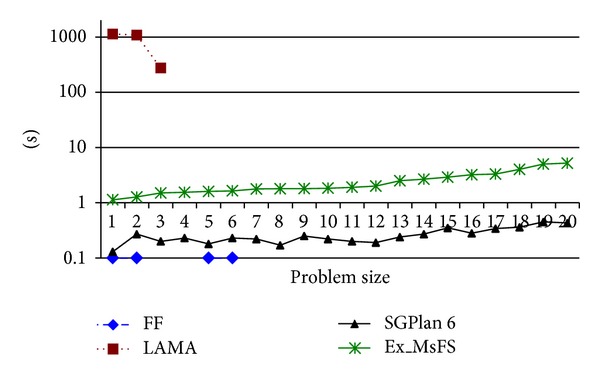
Planning results of the Floortile.

Figure 6 shows the running time curves of the four different search approaches on the Floortile domain. There are 20 instances while LAMA 2008 can only solve 3 instances and FF can only solve 4 instances. However, SGPlan6 and the *Ex_MsFS* algorithm can solve all of the instances. Moreover, in this domain, SGPlan6 can solve each instance with the fewer time cost than *Ex_MsFS*. The reason is that the incremental planning process is very suitable in handling the FGOs of the Floortile domain. Taking the instance displayed in Figure 1 as an example, the incremental planning process first paints the upest tiles as (3,1), (3,2), and (3,3). Then, it paints the tiles (2,1), (2,2), and (2,3). At last, it paints the tiles (1,1), (1,2), and (1,3). This process satisfies the FGO constraints.

For the air defense of naval group problem, SGPlan6 can only solve only 1 out 0f the 17 instances while FF and LAMA 2008 can solve 10 instances. Moreover, as there are time constraints during the air defense process of the naval group in real world, if we require that each instance should be solved within 20 seconds, FF can solve only 7 instances and LAMA 2008 can only solve 6 instances. However, the *Ex_MsFS* approach can solve all of the 17 instances and return the solution plan for each instance within 10 seconds.

**Figure 7:**
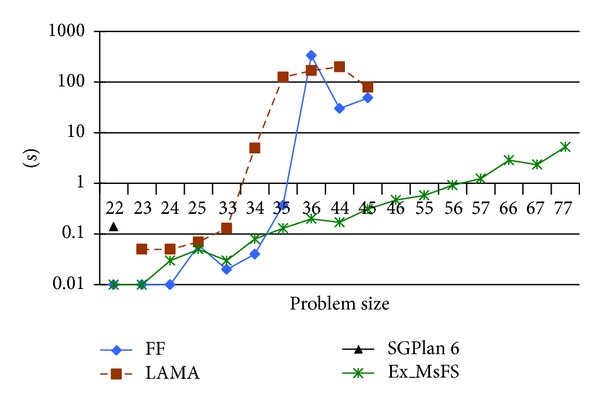
Planning results of air defense of naval group problem.

Based on Figure 7, it can be inferred that air defense of naval group problem is a new challenge for many current planners and *Ex_MsFS* can solve the planning problems with different FGOs efficiently.

## 8. Conclusions and Future Works

This paper contributes to introduce a new planning domain with FGOs, for which all the current related planners do not perform well when solving it. Then, a new search algorithm is proposed which can solve the planning problem with different FGOs efficiently.

For future works, the *Ex_MsFS* should be extended to a more general case to solve more real-world problems with FGOs. Also, machine learning approach [16, 17] can be employed to obtain more informed about FGOs. The learned knowledge can improve the efficiency of the search process. Moreover, a human-computer method is considered for planning with FGO constraints. As some FGO constraints may easily be inferred by humans and other FGO constraints can be learned easily by computers, a human-computer method can be employed in a mixture of automatic and hand-crafted control rules [18]. In addition, if two different domains have similar subgoals interaction, the knowledge of deadlock checking leaned from one domain can be considered transferrable to other domains [19].

## Figures and Tables

**Algorithm 1:**
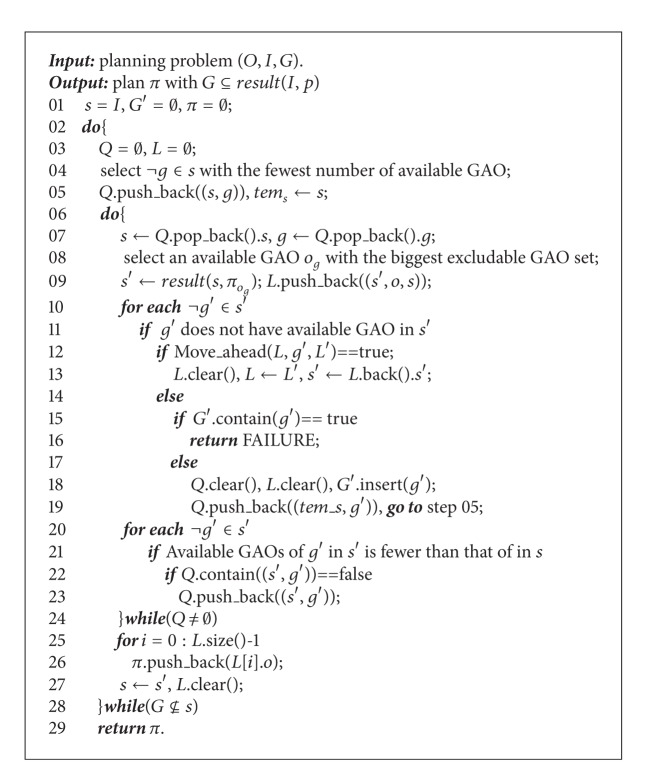
*Ex_MsFS* algorithm.

**Algorithm 2:**
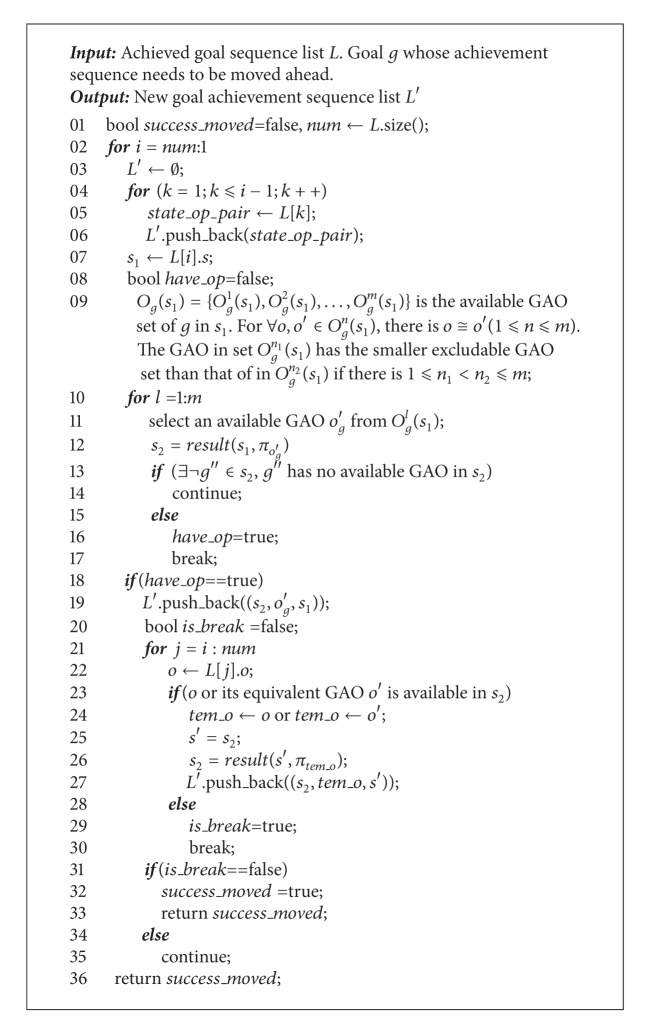
*Mo* *ve*_*ahead*(*L*, *g*, *L*′).
